# Feasibility of recruiting adolescents into a prospective cohort study of the effects of social isolation during COVID-19

**DOI:** 10.1186/s40814-023-01418-8

**Published:** 2023-11-24

**Authors:** Kain Kim, Andrew Jergel, Shasha Bai, Kolbi Bradley, Brianna Karim, Amit Shah, Shakira Suglia, Ngozi Ugboh, Holly C. Gooding

**Affiliations:** 1 Emory University School of Medicine, 2015 Uppergate Dr, Atlanta, GA 30307 USA; 2 Department of Pediatrics, Emory University School of Medicine, 49 Jesse Hill Jr Dr SE, Atlanta, GA 30303 USA; 3 Department of Epidemiology, Emory University Rollins School of Public Health, 1518 Clifton Rd, Atlanta, GA 30322 USA; 4 Ross University School of Medicine, 2300 SW 145th Ave #200, Miramar, FL 33027 USA; 5 Children’s Healthcare of Atlanta, Atlanta, GA USA

**Keywords:** Adolescence, COVID-19, Cohort study, Social isolation, Loneliness, Cardiovascular health

## Abstract

**Background:**

Social connection and loneliness in adolescence are increasingly understood as critical influences on adult mental and physical health. The unique impact of the social isolation imposed by the COVID-19 lockdown on emerging adults is therefore expected to be especially profound. We sought to investigate the feasibility of using ecological momentary assessment (EMA) and wearable accelerometers to characterize the effects of social isolation and/or loneliness experienced by adolescents during the COVID-19 pandemic.

**Methods:**

We recruited 19 participants aged 13–18 from an Adolescent Medicine practice in Atlanta, GA. Participants completed surveys at baseline and throughout a 2-week study period using EMA regarding their degree of social isolation, loneliness, family functioning, school climate, social media use, and COVID-19 experiences surrounding their physical, mental, and social domains. Six participants agreed to wear an activity tracker and heart rate measurement device for 14 days to monitor their emotional state and physical health. Participant feedback was collected via open-ended exit interviews. Feasibility of recruitment/retention, adherence, and outcome measures were investigated. Implementation was also assessed by evaluating the barriers and facilitators to study delivery. Associations between the social isolation and loneliness variables and all other variables were performed with univariate linear regression analysis with significance set at *p* < 0.05. The progression criteria were a recruitment rate of > 30% and a retention rate of > 80%.

**Results:**

Progression criteria were met for recruitment (76%) of participants, but not retention (38%). Adherence to EMA survey completion was highly variable with only 54% completing ≥ 1 survey a day, and accelerometry use was not feasible. Social isolation was significantly correlated with lower school climate, higher COVID-19 experiences, higher depression scores, and lower sleep quality. Loneliness also showed a significant correlation with all these factors except COVID-19 experiences.

**Conclusions:**

EMA and wearable accelerometer use was not feasible in this longitudinal study of adolescents during the COVID-19 pandemic. Future research should further investigate barriers to conducting long-term research with adolescents and the potential effects of the pandemic on subject recruitment and retention.

**Supplementary Information:**

The online version contains supplementary material available at 10.1186/s40814-023-01418-8.

## Key messages regarding feasibility

- What uncertainties existed regarding the feasibility?
  What is the feasibility of using ecological momentary assessment (EMA) and/or wearable biometric tracking devices in adolescent populations during the COVID-19 pandemic? How can we optimize the recruitment and retention of adolescents in a longitudinal study during the COVID-19 pandemic?
- What are the key feasibility findings?
  This feasibility study satisfied the 30% recruitment rate but did not meet the 80% retention rate. Feasibility and accessibility of wearable accelerometers were limited. It was not feasible to provide every participant with a wearable device due to poor return rates.
- What are the implications of the feasibility findings for the design of the main study?
  We recommend that changes to the main study design focus on reducing the burden of data collection, such as decreasing the amount of survey items and optimizing app-based usage of survey reminders. Future methods investigating adolescent heart/physical activity may consider alternative methods to wearable accelerometers for collecting data such as personal smartphones and commercial fitness devices.

## Background

Social connection and support are associated with mental and physical health [1, 2] across the life course. Social connection is especially important during adolescence, a time of intensifying social relationships [3] as well as the emergence of mental health conditions [3] and cardiovascular disease risk factors [4]. The long-term impact of social isolation imposed by the COVID-19 pandemic on adolescents is therefore expected to be profound. Analyses from the United States Centers for Disease Control (CDC) indicate that in 2021, 37% of high school students reported experiencing poor mental health during the COVID-19 pandemic, and 44% reported feeling persistently sad or hopeless in the past year [5]. These changes in adolescent mental health were likely also influenced through interacting effects with various lifestyle changes incited by the pandemic. Though research is ongoing, findings show that the COVID-19 pandemic in the USA has increased rates of obesity, smoking, alcohol consumption, sedentary activity, and screen time [6–8].

Certain environmental factors are likely to have mediated the effects of the pandemic lockdown on adolescent mental health. Adverse childhood experiences (ACES) are known to be associated with poor mental health and suicidal behaviors. In a nationally representative survey of public and private high school students during the COVID-19 pandemic, over half of adolescents (55%) reported experiencing emotional abuse by a parent or other adult in the home, 11% reported physical abuse, and 29% reported a parent or other adult at home lost a job [5]. The prevalence of poor current mental health and suicide attempts in 2021 were 4 and 25 times as high, respectively, for those adolescents with 4 or more ACEs occurring during the pandemic compared to those without [9]. In contrast, research has identified “school connectedness”—the sense of being cared for, supported, and belonging at school—as playing a key protective role in students’ wellbeing during the pandemic. Those who reported more school connectedness were significantly less likely to report feelings of sadness, hopelessness, or suicidal ideation. Unfortunately, less than half (47%) of youth reported feeling school connectedness during the pandemic [5].

The connection between social connection and cardiovascular health in adolescence generally and during the pandemic specifically is less well studied. Studies predating the pandemic have found that people who were socially isolated from their peers as children have less optimal cardiovascular health metrics at 26 years of age [10] and higher levels of inflammation as measured by c-reactive protein in midlife [11]. In a 2022 cohort study of a national sample of 4830 US adolescents, an increase in the Black-White gap in school belonging was associated with an increased risk of diabetes and metabolic syndrome among Black students [12]. Potential mechanisms linking social isolation with cardiovascular health include increased peripheral vascular resistance, increased inflammation, and dysregulated HPA-axis activity [13]. One established mechanism for assessing the impact of these systems on cardiovascular health is heart rate variability (HRV) as it reflects a complex interplay between the physiologic, cognitive, and emotional regulatory systems. In general, greater HRV is associated with a greater capacity to respond to physical and emotional stress. Baseline HRV is reduced in both adolescents and adults with depression [14] and in adolescents with anxiety [15]. HRV in adolescence is associated with other important lifestyle factors related to cardiovascular health including physical activity [16] and sleep duration and efficiency [17, 18].

To better understand the relationships between social connectedness, mental health, and cardiovascular health in adolescence, as well as the COVID-19 pandemic on these relationships, we conducted a feasibility study using ecological momentary assessment (EMA), an activity tracker, and a heart rate monitor to capture real-time data about adolescent behaviors, psychological functioning, and cardiovascular reactivity. Feasibility has been defined as the extent to which a new procedure can be successfully delivered in a distinctive context that is not fully controlled [19]. Other studies have utilized both EMA [20] and activity trackers [21] and found both are feasible with adolescents, with 81% completing up to four surveys per day and 75% of adolescents wearing the activity tracker most days [22], but these were conducted before the COVID-19 pandemic. Other recent feasibility studies employing EMA for daily experience sampling [23] have also found reasonably high rates in daily reporting, though these studies typically target adult populations. Meanwhile, studies requiring participants to wear activity trackers will more often use user-friendly, commercially available products such as Fitbit [24], or even accelerometers worn as an accessory [25]. Few studies have investigated the use of more technically demanding, ambulatory Holter monitors such as the Bittium utilized in our study, especially not in adolescent populations. Therefore, our study addresses a gap in the literature at a time when individuals’ behavioral patterns and lifestyle routines are likely to be impacted in a novel way by the pandemic.

In addition to the physical health measures through the activity tracker, we used EMA to capture adolescent perceptions of social connection, isolation, loneliness, and other emotional states in real time. The COVID-19 experiences (COVEX) scale examines how a variety of mental, physical, and social factors was affected by COVID-19 [26]. Together with surveys of self-reported family functioning, school connectedness, social media use, peer victimization, and discrimination, we hypothesized the EMA and activity tracker would offer a rich contextualization of the impact of social isolation due to the COVID-19 pandemic on adolescent mental and cardiovascular health. Study feasibility was analyzed in accordance with the guidelines from the Consolidated Standards of Reporting Trials (CONSORT) 2010 statement that was extended to include randomized pilot and feasibility studies [27].

## Methods

### Aim, design, and setting

The aims of the study were as follows:

1. To evaluate the feasibility of a prospective cohort study of social isolation in adolescence that utilizes ecological momentary assessment, activity tracking, and heart rate variability.
2. To quantify the effects of social isolation on adolescent physical activity, dietary quality, sleep, and heart rate variability.
3. To identify mediating factors that exacerbate or mitigate the effects of social isolation on adolescents during the COVID-19 pandemic.
4. To assess barriers and facilitators that affect study delivery.

Adolescents ages 13–18 years presenting for a primary care visit at the Children’s Healthcare of Atlanta Adolescent Medicine practice were recruited to participate in this prospective cohort study.

### Participants

Study recruitment took place from January 14, 2022, to July 18, 2022. A trained clinical research coordinator screened for eligible participants using the clinical calendar in the practice Electronic Medical Record system.

Inclusion criteria were as follows:

- Age 13–18 years
- Presents for a primary care visit at the Children’s Adolescent Medicine practice
- Ability to speak, read, and comprehend English

Exclusion criteria are as follows:

- Younger than 13 years or older than 18 years
- Unable to speak, read, and comprehend English
- Known cognitive impairment
- Pregnant women or women who become pregnant
- Refusal or inability to provide consent

Eligible participants were added to the screening log and contacted via phone prior to their visit to see if they were interested in learning more about the study at the time of their visit. Unless a patient declined to learn more about the study at their clinical visit, those presenting in person were approached in the waiting room of the clinic while waiting for their primary care visit to start. Interested patients completed the screening questionnaire to ensure eligibility. Next, the research coordinator explained the study in detail and obtained written informed consent from the guardian (or participant if 18 years of age) and assent from the participant if under 18 years of age. The study overview and informed consent/assent process took place in a private research room of the clinic.

### Study processes

#### Baseline visit

After providing informed consent, participants completed baseline surveys either on a study iPad or via a secure REDCap link sent to the participant’s email and completed on a personal device. The baseline surveys included validated scales of social connectedness [28], loneliness [29, 30], social media use [31], adverse childhood experiences [32], peer victimization, discrimination [33], school climate, family cohesion [34], COVID-19 experiences [35], and sociodemographic factors including gender, race, and ethnicity (see Supplementary Table 1). The mentioned scales were chosen to assess these specific factors based on the availability of published studies on their validity and reliability. Height, weight, and blood pressure measures were extracted from their medical record using the clinical measures obtained during their intake exam on the day they were approached.

Participants then self-selected into one of two arms of the prospective cohort study: (1) wearable devices + EMA surveys or (2) EMA surveys only. Participants in arm 1 were fitted with an ActiGraph GT9X Link with accompanying Polar H7 Bluetooth Heart Rate Monitor. An accompanying manual outlined pertinent information regarding setup, initialization, data collection, and information for subjects. Participants in both arms were instructed on how to download the “RealLife Exp” on their personal device. The RealLife Exp app is an iOS/Android app that sends push notifications to participants’ mobile devices to alert them to complete daily EMA surveys.

#### During study

Participants in arm 1 were monitored at home for daily social connectedness, isolation, loneliness, mood, activity, and autonomic function for 14 days, which is adequate to obtain stable estimates of heart rate and actigraphy indices of behavior based on prior studies [36]. On day 14, subjects were instructed to remove the ActiGraph and Bluetooth monitor and return them using a prepaid package or in-person during their end-of-study visit. Participants in arm 2 were monitored at home for daily social connectedness, isolation, loneliness, and mood, completing EMA surveys only and did not receive the ActiGraph and Bluetooth Monitor.

#### Monitoring of social connectedness, isolation, loneliness, and mood via EMA

Following the approach of the MIDUS II National Study of Daily Experiences (NSDE) [37], we assessed daily social connectedness, isolation, loneliness, and negative/positive affect. All participants randomly received a text message four times per day for 14 days instructing them to complete the EMA surveys via the RealLife Exp app Notifications were pushed to the participants’ phones on a fixed schedule and tapping the notification allowed them to answer questions right away. The app did not collect any PHI and participants were assigned a study ID to provide anonymity to participants when answering questions and to allow the study team to link responses to other data collected on participants throughout the study. The study team was not able to monitor any activity associated with participants’ mobile devices. The platform and data storage protocols for the app are HIPAA compliant. When subjects reached the end of the study duration, they were instructed to remove the app from their phone.

#### Activity tracker

Participants in arm 1 recorded activity and sleep using actigraphy during the 14-day monitoring period. We used the ActiGraph GT9X Link wristwatch-style device containing a calibrated accelerometer that records movement activity in discrete epochs (30 s or 1 min) and detects physical activity as well as the onset and offset of sleep. Sleep parameters were obtained using a scoring algorithm.

#### Heart rate variability

Study staff taught participants in arm 1 how to apply the Polar H7 Bluetooth Heart Rate Monitor during the baseline visit and instructed participants to wear the monitor for up to 14 days, but for at least 24 h. ECG data was collected at 250 Hz sampling frequency and 10-bit resolution. Raw ECG data was sent to an Emory collaborating lab for analysis using custom-built validated software which provided signal quality indices, abnormal rhythm detection, HRV indices, and deceleration capacity.

The CRC assessed the study logs via the activity tracker remote monitoring website daily and contacted participants via text message if their device did not register data for 24 h or no EMA assessments were completed for 24 h.

#### Ongoing monthly surveys

All participants were contacted by the CRC to document their consent to continue to be contacted for monthly surveys administered via a REDCap link sent to the participant’s personal device.

#### Recruitment methods

Participants were compensated $25 for their participation in the baseline study visit, up to $50 for their participation in the home monitoring portion of the study, and $5 for each future monthly survey completed for a total of up to $50 for 10 additional surveys. Participants in arm 1 were compensated up to $50 for completing the EMA surveys and wearing the monitoring devices during the 14-day home monitoring portion. Participants in arm 2 received up to $25 for completing the EMA surveys during the 14-day home monitoring potion. Compensation for participation in the home monitoring portion of the study was dependent on the number of EMAs completed, the proportion of days the activity tracker was worn, and the successful return of the monitoring devices. All compensation was delivered via ClinCard, a reloadable debit card given to the participant at the time of study enrollment, or an Amazon e-gift card. As study procedures were completed, funds were loaded onto the participants’ preferred method of compensation.

### Analytic procedures

#### Feasibility and usability of assessment tools (primary outcomes)

Recruitment rates were calculated as a percentage based on the number of patients initially approached vs. the number of patients who enrolled in the study. Dropout was defined as any patient (at personal or parental request) who chose to no longer submit EMA surveys or did not return the wearable device.

Adherence was operationalized as completion of the baseline survey, completion of at least 4 days of daily EMA surveys (4 surveys sent daily, equating to 56 possible surveys over the course of the 14-day period), successful return of the wearable device by mail (regardless of whether the data met the criteria for usability), and wearing of the ActiGraph device satisfying the usability criteria. The usability criteria for ActiGraph data was defined as at least 8 h of “wear time”, where “wear time” is “non-wear time” subtracted from 24 h. “Non-wear time” was defined as any interval of at least 60 consecutive minutes of zero activity [38, 39].

Semi-structured exit interviews were also conducted to collect qualitative data from participants. The interview questionnaire included four domains relating to subject perceptions of feasibility, barriers, and facilitators of study delivery. The first domain included questions regarding whether participants recalled their participation in the study and what was required of them as a participant. The second domain addressed factors that influenced subject participation in the study, such as facilitators and barriers to EMA survey engagement and compliance with the wearable devices. The third domain focused on factors that hindered device return and the fourth domain gauged subject attitude toward the study. The CRC attempted to contact all thirty participants to conduct these semi-structured interviews. Three total attempts were made to contact participants via phone call. Follow-up was conducted through a 12-month period, concluding on April 16, 2023.

The criteria to assess whether the study should progress to a larger cohort study were a recruitment rate of 30% and a retention rate of 80%. The overall acceptability of the study was evaluated in accordance with the Theoretical Framework for Accessibility framework [40].

#### Potential effects of the COVID-19 pandemic on adolescent health (secondary outcomes)

The potential effects of the COVID-19 pandemic and resulting social isolation on adolescent health factors were examined using baseline survey data. At their baseline study visit, 19 participants completed surveys regarding their degree of social isolation (via the PROMIS Social Isolation Scale), loneliness (via the UCLA Loneliness Scale), family functioning, school climate, social media use, and COVID-19 experiences. We collected available cardiovascular biomarkers including body mass index and blood pressure from the electronic medical record for 18 of the 19 participants. Associations between the social isolation and loneliness variables and all other variables were performed with univariate linear regression analysis with significance set at *p* < 0.05. All statistical analyses were performed in R (version: 4.2.1).

The full trial protocol can be found in the Supplemental materials (feasibility of recruiting adolescents into a prospective cohort study of the effects of social isolation during COVID-19 protocol).

## Results

### Primary outcomes: feasibility and usability of assessment tools

#### Demographics of study participants

Demographic data was collected from 19 participants in the study. Participants ages ranged from 14 to 18 years, and most identified as female and Black/African American. On average, they contributed 12 days of EMA data and 8 days of ActiGraph/HR Monitor Data. Demographic data are presented in Table 1.

**Table 1:** Participant demographics and engagement in the Social Isolation in Adolescent Health (SIAH) study

Characteristic	*N* = 19
**Age,** years	16 (1)
**Gender**	
Female	13 (68%)
Male	6 (32%)
**Race**	
Black/African American	16 (84%)
Hispanic or Latino	2 (11%)
White	1 (5%)
**Days of EMA data (out of a possible 14)**	12 (2)
**Number of EMA Survey Sessions Completed (out of a possible 56)**	30 (12)
**Days of ActiGraph Data (out of a possible 14)**	8 (1)
*No ActiGraph Data*	13
**Days of HR monitor data (out of a possible 14)**	8 (1)
*No HR monitor data*	13

Results are presented as mean (SD) or *n* (%)

### Recruitment and retention

Fifty patients were approached to gauge their interest in participating in the study. Among these, 38 (76%) indicated interest in potentially enrolling. After screening for eligibility criteria, 30 (60%) ultimately provided informed consent/assent and enrolled in the study. Fourteen subjects self-selected to use the wearable activity trackers and were instructed to complete both the baseline surveys and 4 daily EMAs, and the remaining 16 self-selected to only complete the baseline surveys and EMAs.

**Fig. 1:**
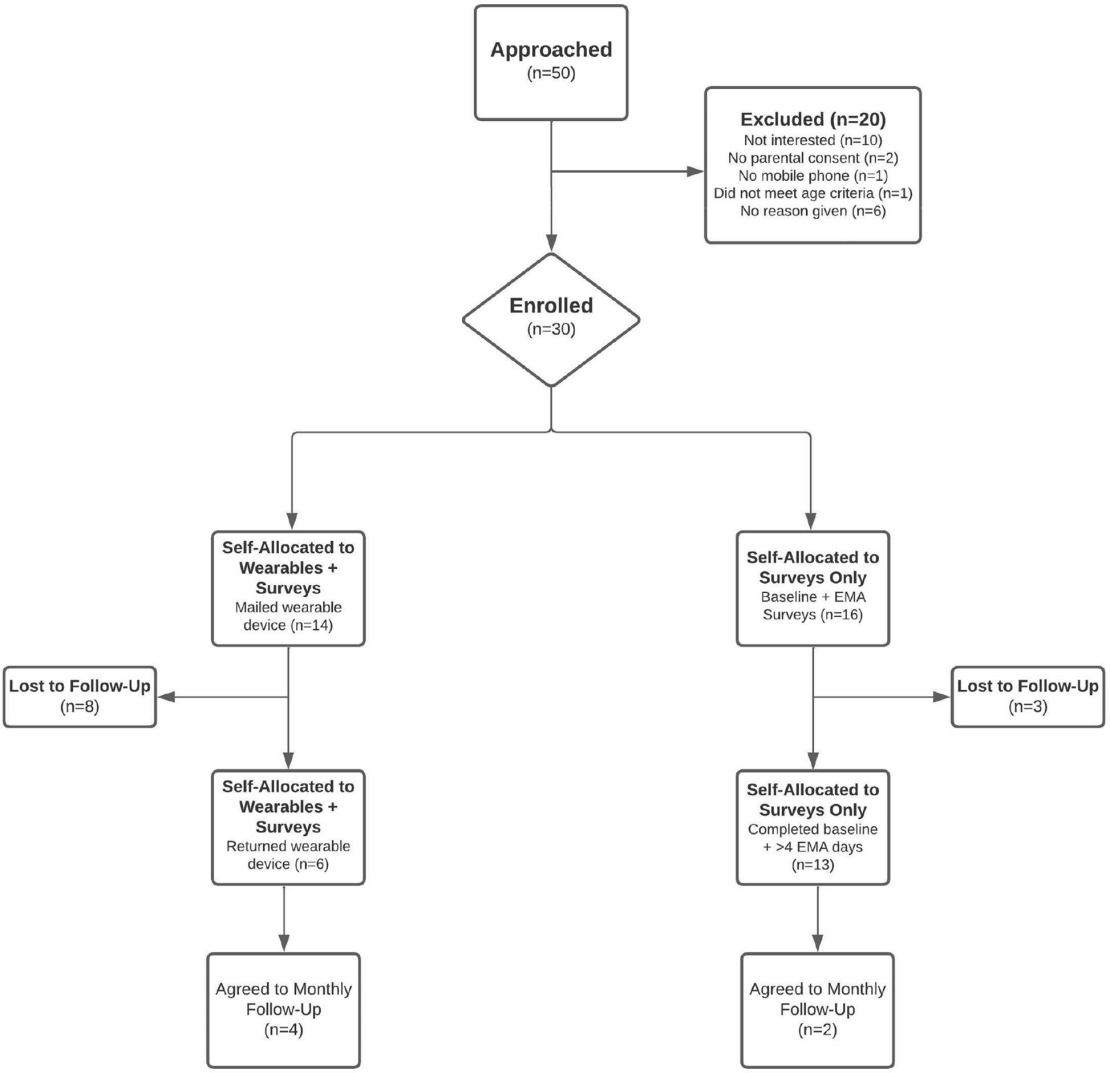
Flowchart representation of participant enrollment and allocation

Eleven participants were discontinued because they failed to meet the threshold for sufficient analyzable data. Four subjects completed neither the baseline survey nor any EMAs, 3 subjects completed at least one EMA but did not complete the baseline survey, and one subject completed the baseline survey but no EMAs. Of the 14 subjects who self-selected to use the wearable activity trackers, only 6 participants returned their devices for data extraction. The remaining 19 participants had sufficient sets of demographic, baseline, and EMA data and were therefore considered eligible for assessing intervention adherence. After concluding the 2-week study period, 9 of the 19 subjects consented when requested to participate in the longitudinal monthly follow-up surveys. Recruitment and retention results are summarized in Fig. 1.

### Usability of outcome measures

**Fig. 2:**
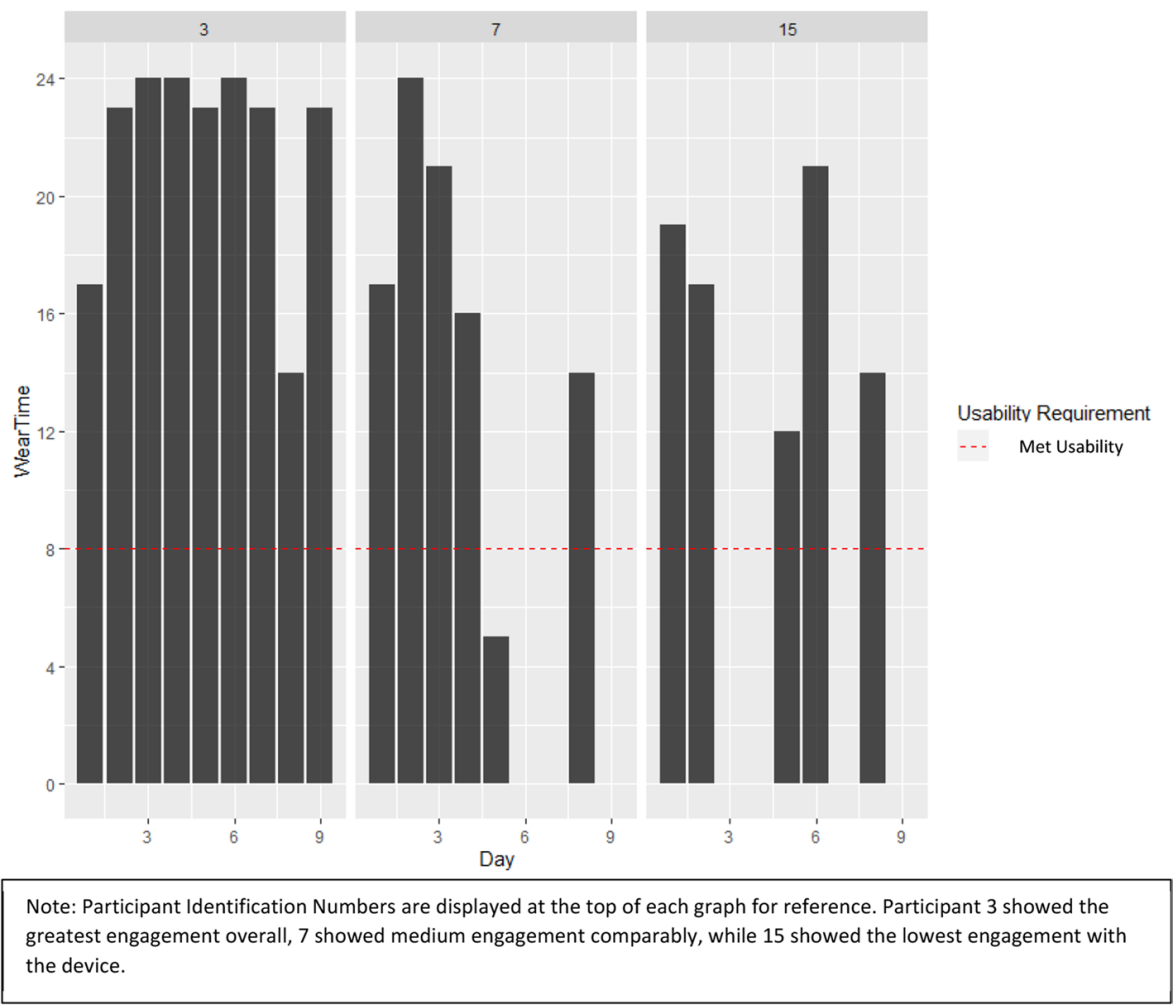
Device usage for three representative ActiGraph participants

Six subjects returned wearable devices, after 14 days of use (84 days total) and contributed 45 days (54%) of ActiGraph data, of which 34 days (40%) met the wearable device criteria of at least 8 h per day of “wear” time. Three participants, who demonstrated different arbitrary levels of engagement, were chosen as examples to show their average daily wear time (Fig. 2). Daily EMA survey completion (fully completed, partially completed, and no attempts) for all 19 subjects who finished the study, are reported in Table 2. Out of the 19 participants, there were 266 days of surveys: 59 days (22%) had 100% completion, 143 days (54%) had at least 1 daily survey completed, and 63 days (24%) had zero attempts. When only considering those who self-selected into the wearables + EMA group, the percentage of EMA completion was relatively the same at 23%, 54%, and 24% respectively.

**Table 2:** Number of completed, partially, and not attempted days of EMA surveys

**Number of eligible survey days**	**Number of times completing all four daily surveys**	**Number of times completing at least 1 of four daily surveys**	**Number of times none of the four daily surveys were completed/attempted**
**Overall, *n* = 19, eligible survey days = 266**	59 (22%)	143 (54%)	63 (24%)
**Only in arm 1 using the wearable devices, *n* = 6, eligible survey days = 84**	19 (23%)	45 (54%)	20 (24%)

The total number of days a participant can complete up to four daily surveys is 14

### Participant feedback during exit interviews

After making three attempts via phone to all enrolled participants (*n* = 30), six participants agreed to participate in the semi-structured exit interview. For the 24 participants who did not participate, 13 were sent voicemails but did not return the calls, 3 had disconnected phone lines, 3 participants requested to be contacted at more convenient times but were unable to be reached at those times, and 2 participants declined to participate with no reason given. Of the 6 participants who agreed to participate in the semi-structured interview, 2 did not recall anything about the study nor what was required of them during their enrollment. The third participant vaguely remembered the study but did recall having to wear wearable devices and complete surveys. They did not recall any positive or unexpected experiences during the study. The fourth participant remembers the study is about “measuring physical and mental health and how they were affected by the pandemic”. They also recalled completing surveys/questionnaires and wearing wearable devices. This participant stopped completing surveys after “losing track of them while being stressed during [their] senior year of high school”, however did note that the study “allowed [them] to be more aware of [their] health”. The fifth participant recalled that the study was about “COVID and how it impacted teenagers” and remembered “answering questions”. The participants completed the surveys until text message reminders stopped being sent to them. They did not have time to engage in the study after “being on the phone a lot with a friend and being busy with school”. The sixth participant remembered answering monthly survey questions, but eventually stopped due to personal health issues. This participant stated that the study allowed them to notice patterns regarding their health habits.

#### Secondary outcomes: potential effects of COVID19 and social isolation on adolescent health

Of the 19 participants who completed the baseline survey, none met the threshold criteria for moderate to severe depression and/or anxiety as described by the PHQ9 and GAD7 assessment tools, respectively. Most participants reported perceiving their social media usage as positive rather than negative (Table 3). We found a significant correlation between social isolation and lower school climate, higher COVID-19 experiences, higher depression scores, and lower sleep quality (Supplemental Table 1). Loneliness showed a significant correlation with lower school climate, higher depression scores, and lower sleep quality, but not COVID-19 experiences (Supplemental Table 2). Family functioning, anxiety, and social media use were not correlated with either social isolation or loneliness.

**Table 3:** Baseline survey data of those with demographic information only (*n* = 19)

**Characteristic**	***N* = 19**^**a**^
**McMaster Family Function Scale** *Possible score range 12–48 (higher score* = *greater functioning)*	24 (8)
**UCLA Loneliness Scale** *Possible score range 20–80 (higher score* = *greater loneliness)*	45 (11)
**PROMIS Social Isolation Scale** *Possible score range 5–20 (higher score* = *greater isolation)*	10 (4)
**PHQ9 Depression score** *Possible score range 0–27 (*> *5* = *mild depression)*	8 (6)
**GAD7 Anxiety score** *Possible score range 0–21 (*> *5* = *mild anxiety)*	2 (1)
**Moderate-Severe depression**	
*Yes*	0 (0%)
*No*	19 (100%)
**Moderate-severe anxiety**	
*Yes*	0 (0%)
*No*	19 (100%)
**% Of time social media positive**	71 (24)
*Missing*	3
**% Of time social media negative**	36 (32)
*Missing*	3
**School Climate Scale** *Possible score range 0–20 (higher score* = *healthier climate)*	13 (4)
**School Victimization Scale** *Possible score range 0–28 (higher score* = *greater bullying)*	10 (5)
**Everyday discrimination scale** *Possible score range 5–30 (higher score* = *greater discrimination)*	12 (7)
**Adverse Childhood Events score** *Possible score range 0–19 (*> *4* = *high number of ACEs)*	4 (4)
*Missing*	3
**High ACE-1 score**	
*Yes*	4 (25%)
*No*	12 (75%)
*Missing*	3
**High ACE-Q score**	
*Yes*	6 (38%)
*No*	10 (63%)
*Missing*	3
**COVEX experiences scale** *Possible score range 1–7 (higher score* = *more negative household experiences)*	5 (1)
**Youth Risk Behavior Physical Activity**	
*Yes*	13 (68%)
*No*	6 (31%)
**Youth risk behavior diet** *Possible score range 0–3 (higher score* = *healthier diet)*	1 (0.4)
**COVEX sleep**	
*Not at all*	7 (39%)
*Several days*	3 (17%)
*More than half the days*	6 (33%)
*Nearly every day*	2 (11%)
*Missing*	1
**COVEX tobacco**	
*Daily or almost every day*	0 (0%)
*3–4 days a week*	1 (5%)
*1–2 days a week*	2 (11%)
*1–3 days a week*	0 (0%)
*Less than once a month*	1 (5%)
*Never*	15 (79%)

Percentages are based on non-missing totals

^a^Mean (SD); *n* (%)

**Fig. 3:**
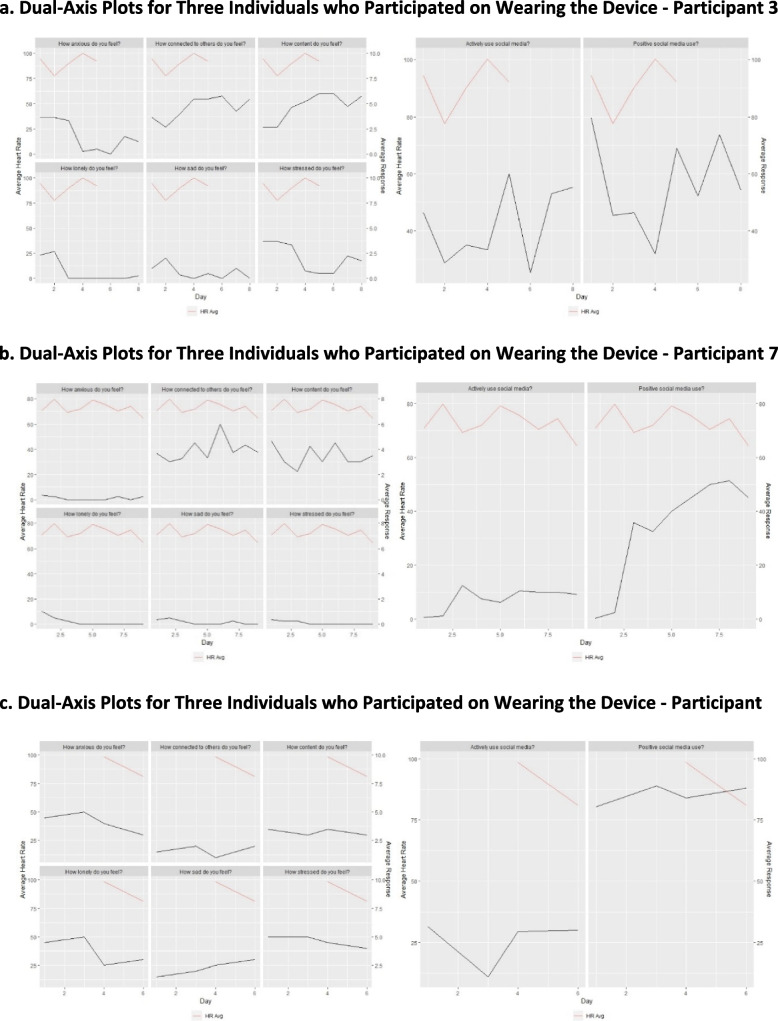
**a** Dual-axis plots for three individuals who participated in wearing the device - participant 3. **b** Dual-axis plots for three individuals who participated in wearing the device - participant 7. **c** Dual-axis plots for three individuals who participated in wearing the device - participant 15

Only one participant had sufficient device usability and survey completion to consistently track their average heart rate and survey responses together. Additional participants were included for comparison (Fig. 3a-c).

## Discussion

Adolescence is marked by rapid psychological change as well as shifts in personal autonomy and social roles. This important developmental period can influence many health determinants, disrupt existing habits, and increase susceptibility to high-risk health behaviors [41]. A study of the impact of COVID-19 on the adolescent period is thus critical to support future public health policy and clinical practice. Longitudinal cohort studies will be essential for mapping any changes in developmental trajectories due to the pandemic. Though all longitudinal research presents significant challenges, working with adolescent and young adult populations introduces its own unique obstacles, often due to the tumultuous nature of this period. Successful longitudinal research is largely dependent on effective recruitment and retention of participants, both of which have been identified as the most challenging aspects of adolescent research [42].

A wide variety of effective adolescent recruitment and retention strategies have been recommended in the literature, such as maximizing the clarity of expectations regarding the study, fostering close cooperation with guardians/school personnel/other stakeholders and staff [43], and maintaining open lines of communication with participants [44]. Importantly, a systematic review and meta-analysis of retention of adolescents in longitudinal cohort studies found that improved retention was associated not with increasing the number of such enhancing strategies, but instead associated with barrier-reduction strategies (i.e., those reducing participant burden, such as flexibility in data collection) [45]. A recent qualitative study of adolescent perspectives on engaging in longitudinal health studies found that key factors for continued involvement include user-friendly reporting (such as technology-based assessment and integration of smartphone apps), flexible scheduling, and avoiding excessive outreach [46].

Although our study did meet the recruitment rate criteria of 30%, our retention rate fell under 80% and was not sufficient to progress to a larger cohort study. Our study did incorporate many of the suggestions for effective research engagement set forth in previous studies, such as app-based data collection, offering financial incentives, and leveraging pre-existing subject relationships with the research team and/or healthcare institution [46]. However, the persistent difficulties in subject retention suggest that there were additional barriers to participation. Two factors likely created further challenges for retaining adolescent participants in this study: the ongoing COVID-19 pandemic itself, as well as belonging to marginalized communities frequently mistrustful of medical research due to a history of unethical practices and racially biased treatment.

Though the challenges presented by working with adolescent and young adult populations have been previously described, the novel limitations presented by the COVID-19 pandemic are less well understood. The restrictions imposed by the pandemic and subsequent lockdown have profoundly disrupted the routines of many children and adolescents, such as by enforcing prolonged co-habitation, increasing the risk of domestic conflict and violence, and reducing support and monitoring from social care services [47]. Moreover, COVID-19 has disproportionately affected minoritized groups such as the population described in this study [48, 49]. It is plausible that a multitude of external demands due to ongoing pandemic effects interfered with participants’ motivation or ability to participate.

In addition, mistrust of healthcare providers and researchers among minoritized individuals presents another significant barrier to study engagement. Research has indicated that mistrust is the most significant barrier to clinical research participation, especially among people from minoritized racial and ethnic groups [50–52]. Histories of negative experiences in healthcare and biomedical research, as well as the resource demand of participation (such as navigating transportation, organizing childcare, and scheduling around work), can make individuals less likely to participate in research studies. While this phenomenon has been less commonly described among adolescents and youth specifically, it is likely that a determinant of adolescent participation is parental distrust, which has been shown to be significantly greater among African Americans [53].

The overall acceptability of the study was evaluated using the Theoretical Framework for Accessibility (TFA) as proposed by Sekhon et al. [40]. The TFA consists of seven constructs: affective attitude, burden, perceived effectiveness, ethicality, intervention coherence, opportunity costs, and self-efficacy. The authors perceive that the acceptability of this study was most compromised by the burden and opportunity costs associated with EMA survey engagement and wearable device adherence. Though only 20% of participants agreed to partake in the post-study interview, those who were responsive generally cited external variables (school stressors, social involvement, personal health matters, etc.) as limiting factors to their completing the study, suggesting time demand (likely attributable to EMAs) was a significant component of the opportunity cost. Notably, half of the responsive participants did not recall specific details about the study during the interview. Given that a third of the respondents agreed the study made them more aware of their health, we do not suspect self-efficacy played a significant role—if anything, it may have raised perceived awareness of personal health patterns. Considering a significant portion of the subjects could not be reached for comment or were unable to recall details about the study, the “intervention coherence”, “perceived effectiveness”, and “affective attitude” constructs in particular may benefit from future exploration. Regarding these areas, a more streamlined and/or detailed enrollment process, as well as closer follow-up throughout the study, may help clarify any existing confusion and/or instill greater motivation to adhere to study terms. A limitation in our analysis according to this framework is our quantitative evaluation: future studies should employ questionnaires or rating scales based on TFA constructs that focus on the anticipated acceptability of the content.

One of the limitations of our study includes the lack of stakeholder engagement such as the use of patient or community advisory boards, who could have facilitated research development and helped participants to better understand the risks and benefits of participation. Moreover, the use of other assessment tools to capture participant experiences may have been better suited for this study’s adolescent population. For example, the Children’s Loneliness and Social Dissatisfaction Scale, which was found to have a clear definition and reliable measure of loneliness, should be considered in future iterations [54]. Finally, the generalizability of the study may be limited by the analysis of a small sample size.

## Conclusions

Despite these challenges, heightened efforts must be made to retain and engage adolescents from vulnerable communities in longitudinal research, especially given the pandemic’s disproportionate impact on minoritized populations. Results from our limited sample suggest that loneliness and social isolation during COVID-19 are associated with many factors in adolescents, with school climate, depressed mood, and sleep quality in particular warranting further study as potential avenues for intervention. Unfortunately, we had too little usable data from the EMAs and wearable devices to draw any further conclusions from this sample. Future studies should identify adolescent-friendly methods that allow for real-time correlation of emotional states, behaviors, physical health metrics, and social contexts in order to best understand the impact of the pandemic on adolescent health.

### Supplementary Information

**Additional file 1: Supplemental Table 1.** Correlation of PROMIS social isolation score and baseline characteristics using univariate linear regression analysis. **Supplemental Table 2.** Correlation of UCLA loneliness scale and baseline characteristics using univariate linear regression analysis.

**Additional file 2.**

## Data Availability

Not applicable.

## Appendix

**Table 4:** Measures used during a prospective cohort study of the effects of social isolation on adolescents during COVID-19

**Baseline**	**During study**	**Ongoing monthly surveys**
UCLA Loneliness Scale [30]	Yung’s Loneliness and Distress Scale 4x/day [55]	UCLA Loneliness Scale
PROMIS Social Isolation Scale [56]	Alber’s Active and Passive Social Media Use Scale 4x/day [57]	PROMIS Social Isolation Scale
General Social Media Use Scale [31]	Social Connectedness Questions 4x/day	General Social Media Use Scale
Georgia Student Health Survey 2.0	Physical Activity via ActiGraph continuously for 14 days – Arm 1 Only	Georgia Student Health Survey 2.0
Everyday Discrimination Scale [33]	Sleep via ActiGraph for 14 days –Arm 1 Only	Everyday Discrimination Scale (Short)
CYW Adverse Childhood Experiences Questionnaire for Adolescents: Self Report [32]	Heart rate variability continuously for at least 24 h and up to 14 days –Arm 1 Only	McMaster Family Functioning Scale
McMaster Family Functioning Scale [34]		COVID-19 Experiences (COVEX)
COVID-19 Experiences (COVEX) [35]		Self-reported physical activity
Self-reported physical activity [58]		Self-reported dietary patterns
Self-reported dietary patterns [58]		Height, weight
Height, weight		Blood pressure
Blood pressure		

## Abbreviations

EMA: Ecological Momentary Assessment
CDC: Centers for Disease Control
ACES: Adverse childhood experiences
HRV: Heart rate variability
COVEX: COVID-19 experiences
NSDE: National Study of Daily Experiences
SIAH: Social Isolation in Adolescent Health
