# Supplementary material for: Feasibility of recruiting adolescents into a prospective cohort study of the effects of social isolation during COVID-19
Source: Pilot Feasibility Stud. 2023 Nov 24;9:191. doi: 10.1186/s40814-023-01418-8 (PMC10668405; doi:10.1186/s40814-023-01418-8)
Supplement: Supplementary file 1 — Additional file 1: Supplemental Table 1. Correlation of PROMIS social isolation score and baseline characteristics using univariate linear regression analysis. Supplemental Table 2. Correlation of UCLA loneliness scale and baseline characteristics using univariate linear regression analysis. [file 40814_2023_1418_MOESM1_ESM.zip › Supplemental Table 1_ESM.docx]

**Supplemental Table 1. Correlation of PROMIS Social Isolation Score and Baseline Characteristics using Univariate Linear Regression Analysis**

| **Characteristic** | **N** | **β** | **95% CI***^1^* **for β** | **p-value** |
| --- | --- | --- | --- | --- |
| **Positive social media use** | 16 | 1.2 | -2.0, 4.4 | 0.483 |
| **GAD7 Anxiety score** | 19 | 0.05 | -0.08, 0.18 | 0.475 |
| **School Climate Scale** | 19 | -0.61 | -1.0, -0.25 | **0.004** |
| **COVID-19 Experiences Scale** | 19 | 0.18 | 0.02, 0.33 | **0.037** |
| **PHQ9 Depression Score** | 17 | 1.1 | 0.56, 1.6 | **0.001** |
| **McMaster Family Function Scale** | 16 | 0.73 | -0.22, 1.7 | 0.154 |
| **Youth Risk Behavior Physical Activity** | 19 | -2.3 | -5.7, 1.1 | 0.207 |
| **Youth Risk Behavior Diet** | 19 | -0.86 | -2.0, 0.32 | 0.172 |
| **Body Mass Index** | 18 | 4.0 | -0.54, 8.5 | 0.103 |
| **Blood Pressure** | 18 | -1.1 | -3.9, 1.6 | 0.420 |
| **Tobacco Use** | 19 | -0.54 | -5.2, 4.1 | 0.823 |
| **Sleep** | 19 | -5.8 | -9.9, -1.8 | **0.012** |
| *^1^*CI = Confidence Interval | | | | |
