# Supplementary material for: Feasibility of recruiting adolescents into a prospective cohort study of the effects of social isolation during COVID-19
Source: Pilot Feasibility Stud. 2023 Nov 24;9:191. doi: 10.1186/s40814-023-01418-8 (PMC10668405; doi:10.1186/s40814-023-01418-8)
Supplement: Supplementary file 2 — Additional file 2. [file 40814_2023_1418_MOESM2_ESM.pdf]

PROTOCOL TITLE: Effects of social isolation on adolescent health during COVID-19

PRINCIPAL INVESTIGATOR:
Holly Gooding, MD, MSc
Section Head for Adolescent Medicine, Department of Pediatrics
Phone: (404) 778-1429
holly.c.gooding@emory.edu

CO-INVESTIGATORS:
Amit Shah, MD, MSCR
Assistant Professor, Department of Epidemiology
Assistant Professor, Department of Medicine, Division of Cardiology
(T) 404-727-8712
ajshah3@emory.edu

Shakira F Suglia, ScD
Associate Professor, Department of Epidemiology
Ph: 404-727-8184
shakira.suglia@emory.edu

RESEARCH COORDINATOR:
Brianna Karim
Section for Adolescent Medicine, Department of Pediatrics, Emory University School of Medicine
407-694-2556
brianna.ann.karim@emory.edu

VERSION: V5, 11.30.2022

FUNDING SOURCE: Emory Adolescent Medicine Departmental Research Funds

REVISION HISTORY

Revision #	Version Date	Summary of Changes
1	10/22/2020	CHOA/Grady Health System language added to consent
2	06/24/2021	Several questionnaires removed; pre-screening language added; screening questions added
3	10/22/2021	Revision of EMA surveys
4	9/29/2022	Added two additional data elements to chart review
5	11/30/2022	Added an over-the-phone/in-person semi-structured interview as a last point of contact to participants

Table of Contents

1. Study Summary 4
2. Objectives 4
3. Background 4
4. Study Endpoints 4
5. Study Intervention/Investigational Agent 4
6. Procedures Involved 5
7. Data and Specimen Banking 6
8. Sharing of Results with Participants 6
9. Study Timelines 6
10. Inclusion and Exclusion Criteria 7
11. Vulnerable Populations 7
12. Local Number of Participants 7
13. Recruitment Methods 8
14. Withdrawal of Participants 8
15. Risks to Participants 8
16. Potential Benefits to Participants 9
17. Data Management and Confidentiality 9
18. Provisions to Monitor the Data to Ensure the Safety of Participants 9
19. Provisions to Protect the Privacy Interests of Participants 10
20. Economic Burden to Participants 10
21. Consent Process 10
22. Setting 13
23. Resources Available 13
24. Multi-Site Research when Emory is the Lead Site 13
25. References 14

1. Study Summary

**Study Title**	Effects of social isolation on adolescent health during COVID-19
**Study Design**	Prospective cohort
**Primary Objective**	To evaluate the feasibility of enrolling adolescents into a prospective cohort study that uses ecological momentary assessment and an activity tracker
**Secondary Objective(s)**	1. To quantify the effects of social isolation on adolescent mental and cardiovascular health metrics including physical activity, dietary quality, sleep, and heart rate variability<br>2. To identify factors that exacerbate or mitigate the effects of social isolation on adolescents during the COVID-19 pandemic
**Research Intervention(s)/Interactions**	Study subjects will complete surveys using ecological momentary assessment and will wear an activity tracker which will measure physical activity, sleep, and heart rate variability
**Study Population**	Adolescents ages 13-18 years of age
**Sample Size**	20
**Study Duration for individual participants**	14 days, with option to continue to be contacted for up to 12 months
**Study Specific Abbreviations/ Definitions**	Coronary Heart Disease (CHD); Cardiovascular Disease (CVD); Clinical Research Coordinator (CRC); COVID-19 Experiences (COVEX); Electrocardiography (ECG); Ecological momentary assessment (EMA); General Anxiety Disorder-7 (GAD-7); Heart rate variability (HRV); Patient Health Questionnaire-9 (PHQ-9); Social Connectedness Scale (SCS);
**Funding Source (if any)**	Gooding Adolescent Medicine Research Fund (internal)

2. Objectives

The purpose of this study is to evaluate the feasibility of enrolling adolescents into a prospective cohort study that uses ecological momentary assessment (EMA) and an activity tracker to quantify the effects of social isolation during the COVID-19 pandemic on mental and cardiovascular health. The objective is to collect pilot data on physical health metrics including physical activity, dietary patterns, sleep, and heart rate variability (HRV), along with measurements of social isolation, social connectedness, and

loneliness. These data will be used to apply for funding for a larger cohort study of the effects of social isolation on adolescent mental and cardiovascular health.

The specific aims are:

A1: Evaluate the feasibility of a prospective cohort study of social isolation in adolescence that utilizes ecological momentary assessment and activity tracking.

H1. A recruitment rate of 30% and a retention rate of 80% would indicate feasibility for a larger cohort study.

A2: To quantify the effects of social isolation on adolescent physical activity, dietary quality, sleep, and heart rate variability. Compared to adolescents who are less socially isolated, adolescents who are more socially isolated will have:

H2a: Lower physical activity as measured by an activity tracker

H2b: Less healthy diets as measured on by self-report

H2c: Later sleep onset and less consolidated sleep as measured by an activity tracker

H2d: Lower heart rate variability as measured by a heart rate monitor

A3: To identify factors that exacerbate or mitigate the effects of social isolation on adolescents. This exploratory aim will measure factors such as self-reported social connectedness, loneliness, social media use, adverse childhood experiences, peer victimization, discrimination, family cohesion, as well as sociodemographic factors including gender, race, family income, parental education, and neighborhood poverty level.

3. Background

Social connection and support are associated with physical and mental health. The mechanisms by which social connection (defined as actual connections to others) and belonging (defined as perceived connections to others), and conversely social isolation (defined as lack of actual connections to others) and loneliness (defined as feelings of sadness due to perceived and actual connections to others), impact health are less well understood. Adolescence is a critical time in the life course for the establishment of mental health, cardiovascular health, and peer social relationships. A more comprehensive understanding of the mechanisms underlying the impact of social connectedness, belonging, social isolation, and loneliness on cardiovascular health during this unique developmental stage is needed. This gap in understanding has only increased in urgency with the COVID-19 pandemic.

Loneliness and social isolation are vitally important in early, middle, and late adolescence and in both girls and boys.[1] Social support and meaningful daily interactions with others are inversely associated with loneliness, with especially strong correlation among young adults.[2] Research indicates that children with persistently high loneliness have more depressive symptoms and lower self-perceived health.[3] The

impact of the social isolation imposed by the COVID-19 pandemic on adolescents is therefore expected to be especially profound. Indeed, psychological distress increased from 3.7% in April 2018 to 24% in April 2020 among young adults ages 18-29 years, and 13.8% of adults reported always or often feeling lonely in April 2020.[4]

In contrast to the data on mental health, the connection between social connection, social isolation, and loneliness and cardiovascular health across the life course is only beginning to be understood. In animal models, socially isolated animals develop more atherosclerosis than those housed in groups.[5] In the few studies done with youth, children who were socially isolated from their peers had less optimal cardiovascular health metrics at 26 year of age[6] and higher levels of inflammation as measured by c-reactive protein in midlife.[7] In adults, social isolation and loneliness are associated with elevated c-reactive protein and later cardiovascular disease (CVD) death,[8] as well as incident CVD events[9] including a 29% increase in risk of incident coronary heart disease (CHD) and a 32% increase in risk of stroke, [10] and future mortality in those with established CVD.[11] Together, these studies establish a relationship between social connectedness, isolation, and loneliness and cardiovascular health while posing important unanswered questions about mechanisms, key developmental periods in the life course, and the impact of unique stressors such as the COVID-19 pandemic.

Potential mechanisms linking loneliness and social isolation with cardiovascular health include increased peripheral vascular resistance, increased inflammation and dysregulated HPA-axis activity.[5] One established mechanism for assessing the impact of all of these systems on cardiovascular health is heart rate variability (HRV) as it reflects a complex interplay between the physiologic, cognitive, and emotional regulatory systems. In general, greater HRV is associated with greater capacity to respond to physical and emotional stress. HRV changes during adolescence, from the relatively higher HRV of childhood to the relatively lower HRV of adults.[12]

HRV has been associated with both mental health and cardiovascular health outcomes. Baseline HRV is reduced in both adolescents and adults with depression[13] and in adolescents with anxiety.[14] Negative social interactions decrease HRV to a similar degree as the Trier Social Stress Task,[15] and social isolation is associated with reduced HRV in adult women.[16] Important developmental differences in HRV in response to stressors may occur. For example, animal models show a greater impact of social isolation on adolescent cardiovascular reactivity as compared to adult rats.[17] In adults with depression, HRV is further blunted after stressful tasks but in adolescents with depression, HRV may actually increase after stressful tasks.[18] Finally, HRV in adolescence is associated with other important lifestyle factors related to cardiovascular health including physical activity[19] and sleep problems[20] such as variable sleep duration and efficiency.[21]

The figure below, from Holt-Lundstead and Smith, describes pathways linking social connection with cardiovascular health through mechanisms such as lifestyle factors, psychological factors, and biomarkers.[22] In this proposal, we propose a feasibility study using ecological momentary assessment (EMA) and an activity tracker to understand the

effect of social isolation in real-time on adolescent behaviors/lifestyle, psychological functioning, and biomarkers relevant to cardiovascular health. This proposal is especially relevant now as adolescents are socially isolated to various degrees during the COVID-19 pandemic.

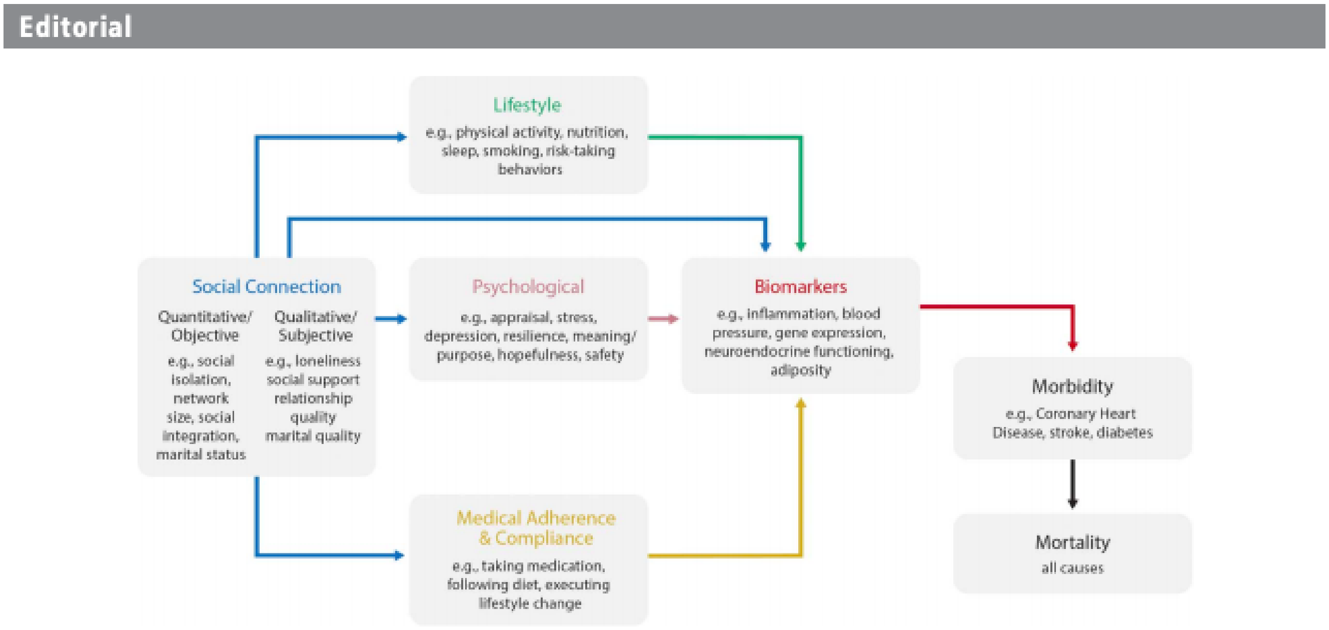

Figure 1 Simplified model of possible direct and indirect pathways by which social connections influence disease morbidity and mortality.

Smaller studies have utilized both EMA[23] and activity trackers and found both are feasible with adolescents, with 81% completing up to four surveys per day and 75% of adolescents wearing the activity tracker most days.[24] A systematic review found wearable activity trackers are generally acceptable to adolescents in research studies,[25] with wrist devices are more acceptable than hip and chest devices to teens.[26] In addition to the physical health measures through the activity tracker, we will use EMA to capture adolescent perceptions of social connection, isolation, loneliness, and other emotional states in real-time. The most widely used measure of loneliness is the UCLA loneliness scale, which has been validated in adolescents ages 12-21.[27,28] We will use this as well as the Social Connectedness Scale (SCS)[29] and the PROMIS social isolation scale.[30] Together along with self-reported social media use, peer victimization, and discrimination, the EMA and activity tracker will allow for a rich contextualization of the impact of degrees of social isolation due to the COVID-19 pandemic on adolescent mental and cardiovascular health.

4. Study Endpoints

The primary study endpoints are 1) feasibility (recruitment and retention rate) and 2) usability (% of days activity tracker is worn, % of EMA surveys completed, % of data that is interpretable). Secondary endpoints include objectively documented physical activity, sleep, self-reported diet, and heart rate variability.

5. Study Intervention/Investigational Agent

Participants will be fitted with an ActiGraph GT9X Link with accompanying Polar H7 Bluetooth Heart Rate Monitor. ActiGraph's activity and heart rate monitors are not subject to FDA regulation as they are not being used to diagnose or treat disease and are not introducing energy into the person's body. An accompanying manual will outline pertinent information regarding setup, initialization, data collection, and information for subjects. The manual has been uploaded with this submission in the "Other Documents" section (ActiGraph_Link_UserGuide.pdf).

Baseline surveys will collect data on validated scales of social connectedness, loneliness, social media use, adverse childhood experiences, peer victimization, discrimination, school climate, and family cohesion; physical and mental health including PHQ-9 and GAD-7; COVID-19 experiences; sociodemographic factors including gender, race, ethnicity, family income, and parental education; neighborhood poverty level and neighborhood-level movement/isolation via geocoding. EMA will use 4 surveys per day delivered via text to collect data on perceived social isolation, loneliness, and social media use. Survey responses will not be audio or video recorded for any participants.

6. Procedures Involved

Study Schema

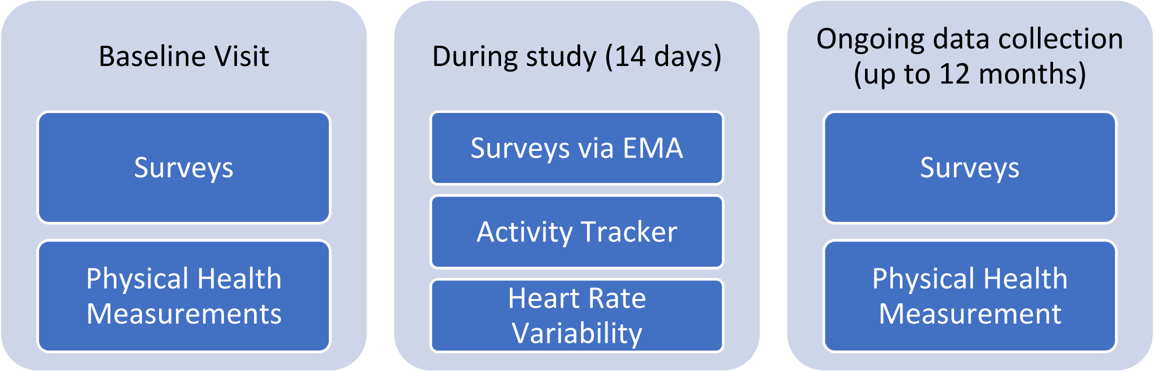

Baseline Visit. After providing informed consent, participants will complete the baseline study visit either in the CHOA Adolescent Research Room (in-person recruitment) or at home via Zoom (telehealth recruitment). The clinical research coordinator (CRC) will administer all baseline surveys via RedCAP either on a study iPad (in-person recruitment) or via a secure link sent to the participant's email. Participants will be taught how to apply the activity tracker and heart rate monitor. Height, weight, blood pressure, non-high density lipoprotein (non-HDL) cholesterol, and glycosylated hemoglobin (HbA1C) will be extracted from the medical record for baseline visits. For subjects approached about the study in person – regardless of if they complete their baseline visit at the clinic or at a later time via Zoom – their height, weight, blood pressure, non-high density lipoprotein (non-HDL) cholesterol, and glycosylated hemoglobin (HbA1C) measures will be extracted from their medical record using the clinical measures obtained during their intake exam on the day they were approached. For subjects

who enroll via telemedicine visits, their height, weight, blood pressure, non-high density lipoprotein (non-HDL) cholesterol, and glycosylated hemoglobin (HbA1C) measures will be extracted from their medical record using the clinical measures obtained during their intake exam on their most recent visit.

During Study. Participants will be monitored at home for daily social connectedness, isolation, loneliness, mood, activity, and autonomic function for 14 days, which is adequate to obtain stable estimates of heart rate and actigraphy indices of behavior based on prior studies.[31] On day 14, subjects will remove the Actigraph and Bluetooth monitor and return them to us using a prepaid package or in-person during their end-of-study visit.

Monitoring of Social Connectedness, Isolation, loneliness, and Mood via EMA. Following the approach of the MIDUS II National Study of Daily Experiences (NSDE),[32] we will assess daily social connectedness, isolation, loneliness and negative/positive affect. Participants will randomly receive a text message four times per day for 14 days instructing them to complete the EMA surveys use an app called "RealLife Exp" which they will be instructed to download on their phone at the baseline visit. The RealLife Exp app is an iOS/Android app that will push notifications to participants' mobile devices to alert them to complete daily surveys. Notifications will be pushed to the participants phone on a fixed schedule and participants will tap the notification allowing them to answer questions right away. The app will not collect any PHI and participants will be assigned a study ID which will help provide anonymity to participants when answering questions and allow the study team to link responses to other data collected on participants throughout the study. The study team will not be able to monitor any activity associated with participants' mobile devices. The platform and data storage protocols for the app are HIPAA compliant. When subjects reach the end of the study duration, they will be instructed to remove the app from their phone. The research coordinator will walk participants through this process.

Participants will be sent a survey derived from the loneliness and distress questionnaire used by Yung et al[34], and containing questions surrounding overall connectedness. This survey will be sent to participants 4 times a day and will ask participants to rate these items based on their experiences over the past few hours. The survey will also contain questions surrounding passive and active social media use[35] since social media has been associated with affect[36] and social media use[37] in youth and may or may not mitigate social isolation due to COVID-19.

Activity Tracker. Participants will record activity and sleep using actigraphy during the 14-day monitoring period. We will use the ActiGraph GT9X Link wristwatch-style actigraph containing a calibrated accelerometer that records movement activity in discrete epochs (30 s or 1 min) and detects physical activity as well as onset and offset of sleep. Sleep parameters are obtained using a scoring algorithm. Subjects will use the event marker on the ActiGraph to record any stressful events and will also keep a written log of these events, their intensity and triggers.

HRV. We will use the Polar H7 Bluetooth Heart Rate Monitor. Study staff will teach the participant how to apply the monitor during the baseline visit and instruct participants to wear

the monitor for up to 14 days, but for at least 24 hours. ECG data will be collected at 250 Hz sampling frequency and 10-bit resolution. We will not collect any PHI with the heart rate monitors. Instead, we will assign participants a study ID and use this for identification purposes. Raw ECG data will be sent to an Emory collaborating lab for analysis using custom-built validated software which will provide signal quality indices, abnormal rhythm detection, HRV indices and deceleration capacity.

The CRC will access the study logs via the activity tracker remote monitoring website daily and contact participants via text message if their device does not register data for 24 hours or no EMA assessments are completed for 24 hours.

Study End. Participants will be contacted by the CRC to document their consent to continue to be contacted for monthly surveys administered via use the “RealLife Exp” application. Participants who do not wish to continue will be instructed in how to remove the application from their phone. Participants who decide to be contacted for monthly surveys will continue to use the RealLife Exp app and receive notifications to complete EMA surveys once per month. After one year of follow-up for those consenting to the monthly EMA surveys, participants will be instructed in how to remove the application from their phone. No data will be collected after 1 year. Participants will be contacted via phone once more by the CRC for a semi-structured interview about their perspective on the study and its requirements. The CRC will also access their chart to view the date and time of their future appointments in the Adolescent Clinic at Children’s Healthcare of Atlanta at Hughes Spalding. Once participants come into the clinic for their appointment, the CRC will approach them to gauge their interest in the interview. If participants agree to participate in the interview, their answers will be collected as part of assessing the feasibility of the study.
All participants will be given a pre-addressed, stamped envelope in which to return the activity tracker and heart rate monitor after the 14-day study duration. No additional data will be collected from participants using these devices after the 14-day period.

Study Measures (see Appendix for details of specific measures)

**Baseline**	**During Study**	**Ongoing Monthly Surveys**
UCLA Loneliness Scale[28]	Yung’s Loneliness and Distress Scale 4x/day[34]	UCLA Loneliness Scale
PROMIS Social Isolation Scale[30]	Alber’s Active and Passive Social Media Use Scale 4x/day[35]	PROMIS Social Isolation Scale
General Social Media Use Scale[37]	Social Connectedness Questions 4x/day	General Social Media Use Scale
Georgia Student Health Survey 2.0	Physical Activity via Actigraph continuously for 14 days	Georgia Student Health Survey 2.0
Everyday Discrimination Scale[39]	Sleep via Actigraph for 14 days	Everyday Discrimination Scale

CYW Adverse Childhood Experiences Questionnaire for Adolescents: Self Report[38]	Heart rate variability continuously for at least 24 hours and up to 14 days	CYW Adverse Childhood Experiences Questionnaire for Adolescents: Self Report
McMaster Family Functioning Scale[40]		McMaster Family Functioning Scale
COVID-19 Experiences (COVEX)[41]		COVID-19 Experiences (COVEX)
Self-reported physical activity[43]		Self-reported physical activity
Self-reported dietary patterns[43]		Self-reported dietary patterns
Height, weight		Height, weight
Blood pressure		Blood pressure

7. Data and Specimen Banking

All data collected from the activity tracker, heart rate monitor, and EMA assessments will be stored until the completion of data analysis, submission of the next grant, and presentation at academic meetings and journals. We anticipate that this will be 21 months from the study start, which includes additional time needed for participants who agree to participate in monthly survey follow-ups for one year. Only study personnel will have access to the data. We will not release the data to other individuals or research groups.

The data to be stored includes survey responses, sleep and activity data, and heart rate data for each participant.

8. Sharing of Results with Participants

Participants will be informed during the consent process that the research team will be monitoring their activity tracker, heart rate monitor, and EMA assessments for the purposes of research only and that most findings will not be disclosed. The exception will be for clinically significant arrythmias detected on the heart rate monitor or for severe depression or anxiety noted during the baseline or follow-up visit. Participants may not opt-out of being contacted if these are noted. Clinically significant arrhythmias that are incidentally found will be reported to the participant, who may elect to share this with his/her physician. Participants with a score in the range of severe depression (PHQ-9 greater than or equal to 15 on the COVEX questionnaire) or anxiety (GAD-7 greater than or equal to 15 on the COVEX questionnaire) during the baseline or follow-up visit will be contacted by the CHOA Adolescent Medicine psychotherapist via telephone. If a participant notes suicidal ideation

9. Study Timelines

Participants who *do not* consent to monthly follow-up surveys following initial study participation will be enrolled in the study for a duration of three months. We anticipate study enrollment to take three months and data analysis/manuscript preparation to

take an additional three months. The total time to study completion is estimated to be nine months.

Participants who *do* consent to monthly follow-up surveys following initial study participation will be enrolled in the study for a duration of 15 months (three months for initial study duration and 12 additional months for monthly survey follow-up). We anticipate study enrollment to take three months and data analysis/manuscript preparation to take an additional three months. The total time to study completion is estimated to be 21 months.

10. Inclusion and Exclusion Criteria

Adolescents ages 13-18 years presenting for a primary care visit at the CHOA Adolescent Medicine practice will be recruited to participate in the study. The research coordinator will screen for eligible participants using the clinical calendar in the Epic Electronic Medical Record system.

Inclusion criteria are as follows:

- Age 13-18 years
- Presents for a primary care visit at the CHOA Adolescent Medicine practice
- Ability to speak, read, and comprehend English

Exclusion criteria are as follows:

- Younger than 13 years or older than 18 years
- Unable to speak, read, and comprehend English
- Known cognitive impairment
- Pregnant women or women who become pregnant
- Refusal or inability to provide consent

11. Vulnerable Populations

The proposed study involves the vulnerable population of children under the legal age of consent to procedures involved in the research. While the research involves greater than minimal risk to children presented by a monitoring procedure which is not likely to contribute to the well-being of the subject, we will take safeguards to protect the rights and welfare of these individuals. Written parental permission will be obtained for all individuals under the age of 18 years. Permission of one parent is sufficient even if the other parent is alive, known, competent, reasonably available, and shares legal responsibility for the care and custody of the child. Only parents or legal guardians listed in the child's medical record will be able to provide permission. Additionally, we will obtain written assent from all children to ensure they understand the study and agree to participate. Because participants may receive clinical care from the PI, who is a provider at the CHOA Adolescent Medicine practice, the consent and assent process will be carried out by the research coordinator. This will aid in minimizing coercion or undue influence by the PI.

12. Local Number of Participants

A total of 20 participants will be recruited for the study. All participants will be recruited from the CHOA Hughes Spalding Adolescent Medicine Practice. Using the social isolation questions found in the screening survey (ATTACHED), we will split participants into two groups for comparison: socially isolated (n=10) vs non-socially isolated (n=10)

13. Recruitment Methods

Adolescents ages 13-18 years presenting for a visit (either in-person or telemedicine) at the CHOA Adolescent Medicine practice will be recruited to participate in the study. The research coordinator will screen for eligible participants using the clinical calendar in the Epic Electronic Medical Record system. Eligible participants will be added to the screening log (ATTACHED) and contacted via phone prior to their visit to see if they are interested in learning more about the study at the time of their visit. Unless a patient declines to learn more about the study at their clinical visit, those presenting in-person will be approached in the waiting room of the clinic while waiting for their primary care visit to start. Interested patients will complete the screening questionnaire (ATTACHED) to ensure eligibility. Next, the research coordinator will explain the study in detail and obtain written informed consent/assent. The study overview and informed consent/assent process will take place in a private research room of the clinic. Alternatively participants will have the option to complete the study enrollment and consent/assent with legal e-signature via REDCap if they prefer to leave the clinical environment after their appointment.

Teens presenting via telemedicine will be asked by their physician if they are interested in learning about the study. If so, they will be informed that they will receive a follow-up call by the research coordinator to schedule a time to discuss the study via Zoom. During the scheduled Zoom videoconference, the research coordinator will explain the study in detail and obtain electronic informed consent/assent with legal e-signature via REDCap.

Participants will be compensated $25 for their participation in the baseline study visit, up to $50 for their participation in the home monitoring portion of the study, and $5 for each future monthly survey completed for a total of up to $50 for 10 additional surveys. Compensation for participation in the home monitoring portion of the study will be dependent on the number of EMAs completed, proportion of days the activity tracker is worn, and successful return of the monitoring devices. All compensation will be delivered via ClinCard, areloadable debit card that will be given to the participant at the time of study enrollment. As study procedures are completed, funds will be loaded onto the participants' card.

14. Withdrawal of Participants

Participants will be told that they can drop out of the study at any time during the study. Participants who become pregnant or become cognitively impaired throughout study duration will be withdrawn from the study without their consent. Furthermore, participants who become otherwise unable to participate in the research procedures due to health-related concerns will be withdrawn from the research without their consent at the discretion of the PI. Data collection will cease for participants who

withdraw from the research and these individuals will not be included in the final data analysis.

15. Risks to Participants

The proposed study involves greater than minimal risk to children presented by a monitoring procedure which is not likely to contribute to the well-being of the subject. A potential risk/discomfort from participation in this study is the use of wearable devices. These devices may be uncomfortable or inconvenient for participants to wear for extended periods of time. Additionally, participants may feel uncomfortable or embarrassed when asked questions related to social connectedness, loneliness, social media use, adverse childhood experiences, peer victimization, and discrimination. To minimize these risks, participants will be given demonstrations on how to appropriately wear the devices and will be able to decline to answer questions they do not feel comfortable answering. Furthermore, participants may withdraw from the study at any time.

Another risk to participants is breach of confidentiality. To ensure participant confidentiality, study identification numbers will be assigned to each participant and used throughout the duration of the study when referring to subjects. The codes that link the name of the participant and the study identification number will be kept confidential by the PI in a secured cabinet. Only the authorized study team with appropriate training will have access to the data.

16. Potential Benefits to Participants

Potential benefits to participants include increased awareness of personal health metrics (i.e. heart rate, physical activity, sleep) as reported by the activity tracker used in this study.

17. Data Management and Confidentiality

Given our proposed sample size of 20 participants, we have at least 80% power to detect a 0.53 standard deviation change from baseline to 3 months on a given outcome measure using a two-sided paired t-test with an alpha = 0.05. We will report descriptive statistics for the EMA assessments. Paired t-tests (or if data are not normally distributed, the Wilcoxon signed-rank test) will be used to compare the baseline and monthly survey data for the continuous scales. Results will be presented as difference in means or medians with associated 95% confidence intervals. McNemar's test for correlated proportions will be used to compared binary outcomes including the baseline and monthly follow up survey data. Effect sizes will be calculated for all measures to further quantify the degree of efficacy. A recruitment rate of 30% and retention rate of 80% would indicate feasibility for a larger trial. Statistical analysis will be conducted using SAS v. 9.4 (Cary, NC) and significance will be assessed at the 0.05 level.

All questionnaire data will be collected using REDCap software. Data related to wearable devices will be collected using the manufacturer's software. To maintain participant confidentiality, all participants will be assigned a study identification number. The codes

that link the name of the participant and the study identification number will be kept confidential by the PI in a secured cabinet.

Upon data storage, all data will be de-identified. Only the authorized study team on this proposal will have access to the data. All study staff will complete biomedical or socio-behavioral training through the Collaborative IRB Training Initiative Program (CITI). We will store the data on the Emory server. The server is protected by the Emory network, and a secured log-in is required from all users. Data will be stored only until final analysis and publication of any manuscripts, after which the data files will be removed. No data will be released to other agencies.

To ensure data quality, the research team will use REDCap validation methods throughout surveys to ensure that entered values are within the specified range and that items are not inappropriately skipped. Data from the wearables will be closely monitored by the study team on a weekly basis to ensure accuracy. Additionally, the research coordinator will routinely check in with the participant to ensure that they are wearing the devices correctly and answer any questions. All participants will receive a through demonstration on how to sync, wear, and charge devices.

18. Provisions to Monitor the Data to Ensure the Safety of Participants

We will establish a Data and Safety Monitoring Plan that consists of the PI and co-investigators. In order to ensure the integrity and validity of the data, Dr. Gooding will conduct a routinely review of reports that describe study operations and present data to ensure that problems are identified and corrected in a timely manner. The following reports will be generated for routine review:

- Screening, recruitment, enrollment, and retention reports
- Protocol compliance reports to identify protocol deviations
- Data quality reports – describe missing, erroneous, and inconsistent data to ensure protocol is followed and deviations are tracked
- Identification/reporting of serious adverse events

If data quality and integrity issues are uncovered, the following correction processes will occur:

- If the quality of the data compromised, an audit of all related data will be conducted by Dr. Gooding.
- If systematic errors are uncovered, Dr. Gooding will review these errors and update the protocol (with IRB approval) to address the cause of the systematic errors.
- If detection processes are not adequately discovering issues related to data quality, new processes will be developed to adequately detect data quality issues.
- If systematic protocol violations are occurring, staff will be retrained by Dr. Gooding.

Diligent safety monitoring will be conducted throughout this study in compliance with the following required elements of the Emory IRB's continuing review process:

1. tracking of subject accrual (enrollment, drop-outs, demographics)
2. timely and appropriate reporting of informed consent process deficiencies, protocol deviations, privacy breaches, conflicts of interest, and/or changes in personnel
3. ongoing monitoring and appropriate reporting of adverse event activity including:

   a. frequency of unanticipated, internal, related or possibly related, and serious or more prevalent than expected adverse events
   b. frequency of deaths occurring during the study or within 30 days of study termination, even if expected or unrelated
4. interim assessment of risk/benefit relationship in reference to adverse event occurrences, preliminary observations, and emerging information
5. timely and appropriate IRB submission of safety-related documents such as audit reports, sponsor progress reports, and other materials or communications that might impact the safe conduct of this study
6. active cooperation with the IRB and other applicable entities in the event of a random or for-cause internal or external audit

The PI and co-investigators will meet monthly to review data quality and safety events, including any protocol deviations, and to adjudicate adverse events. Additionally, participant safety monitoring will occur at each follow-up study visit.

19. Provisions to Protect the Privacy Interests of Participants

Participants will interact with the research coordinator during both the informed consent process and in data collection. Both of these tasks will be carried out in a private research room. There will be no other individuals interacting with or observing the participants.

The PI, who may be familiar with participants from their time receiving treatment in the clinic, will also interact with participants to answer study-related questions as needed. All questions will be answered in a private setting in order to reduce the risk of observation by anyone not participating in the research.

Only the PI and research coordinator will be able to access the participants' medical for the sole purpose of determining eligibility. Only the authorized study team on this proposal will have access to study-related data. All study staff will complete biomedical or socio-behavioral training through the Collaborative IRB Training Initiative Program (CITI). Data will be stored on the Emory servers in password-protected files.

20. Economic Burden to Participants

Individuals will not incur any medical costs by participating in the proposed study. Participants will be provided with all study devices. Participants will need to have their own smartphone with data plan to participate; participants will be provided $25in the form of a gift card to offset additional data usage when completing the EMA, in addition to the compensation for study completion as outlined in Section 13.

21. Consent Process

Written informed consent or electronic consent with legal e-signature (for telemedicine/virtual enrollment) will be obtained for all individuals on the same day as initial data collection. Electronic consent/assent will be obtained via REDCap, which will allow to study team to obtain a legally binding electronic signature. Individuals who are 18 years old will provide written/electronic consent themselves. For individuals younger

than 18 years, parental permission will be provided by the parent or legal guardian. In addition, written/electronic assent will be obtained for all individuals under 18 years. For individuals presenting in-person, eligible patients will be approached by the research coordinator in the waiting room of the CHOA Adolescent Medicine Practice. Patients will be asked if they are interested in learning more about the study. If so, the eligible patient will be led by the research coordinator to the research room of the CHOA Adolescent Medicine practice, which is a private office within the clinic. The research coordinator will then carry out the consent/assent process with the patient and his/her parent or legal guardian. For individuals presenting via telemedicine/virtually, they will be asked by their physician if they are interested in learning about the study. If so, they will be informed that they will receive a follow-up call by the research coordinator to schedule a time to discuss the study via Zoom. During the scheduled Zoom videoconference, the research coordinator will carry out the consent/assent process with the patient and his/her parent or legal guardian. The consent process will take approximately 15 minutes. If the eligible participant or the parent/legal guardian has any questions about the study, they will be answered at this time. In order to minimize the possibility of coercion or undue influence, the consent process will be carried out by the research coordinator, who has no influence on or connection to the medical care received by the patients. The research coordinator will not obtain a signature for consent until the entire study has been explained and all questions have been answered.

Non-English-Speaking Participants

The data collection instruments used in the study will be written in English. Therefore, it will be inappropriate to include subjects who are not proficient in English in this pilot study. This is not to say that the research topic does not apply to adolescents who are not proficient in English; however addressing these populations within the proposed pilot study would not be possible.

Participants who are not yet adults (infants, children, teenagers)

Prospective participants who have not attained the legal age for consent (e.g. individuals under the age of 18 years), as determined by the age in their medical record, will be required to give written/electronic assent. Written/electronic parental permission will also be obtained for these individuals. Parental permission will be obtained from one parent, even if the other parent is alive, known, competent, reasonably available, and shares legal responsibility for the care and custody of the child. Only parents or legal guardians listed in the child's medical record will be able to provide permission.

22. Setting

The research team will recruit 20 patients at the Children's Healthcare of Atlanta (CHOA) Hughes Spalding Adolescent Medicine practice. Patients will be screened for eligibility at the time of their visit. Eligible teens presenting to the clinic in-person will be approached by the research coordinator in the waiting room to see if they would be interested in

learning more about the study. If so, the research coordinator will lead the patient to a private research room in order to provide an overview of the study, carry out the consent/assent process as described in Section 21, and carry out all study procedures. Alternatively, participants will have the option to complete the study enrollment and consent/assent virtually with legal e-signature via REDCap if they prefer to leave the clinical environment after their appointment. Teens presenting via telemedicine will be asked by their physician if they are interested in learning about the study. If so, they will be informed that they will receive a follow-up call by the research coordinator to schedule a time to discuss the study via Zoom. During the scheduled Zoom videoconference, the research coordinator will explain the study in detail, carry out the consent/assent process as described in Section 21, and carry out all study procedures.

23. Resources Available

The CHOA Hughes Spalding Adolescent Medicine Practice serves approximately 2000 adolescents ages 13-21 years of age annually for primary care wellness visits, as well as consultative behavioral/mental health and reproductive health visits. For the proposed study, we will need to recruit 20 participants ages 13-18 years in a two-month time span. The Adolescent Medicine Practice sees patients three days out of the week. We will devote each of those three days to recruitment in order to reach our recruitment goal.

Both medical and psychological resources are available to participants who experience anticipated consequences of human research. Dr. Gooding, the project PI, is a provider at the CHOA Adolescent Medicine practice and is able to provide research-related care to patients as needed. The Adolescent Medicine practice also has two additional medical doctors, a nurse practitioner, and a licensed psychologist who will be made aware of the research study and will be able to provide care as needed to patients experiencing any stress identified via the research protocol.

All persons assisting with the research will be adequately informed about the protocol, research procedures, and their duties and functions. Furthermore, all persons assisting with the research will receive appropriate CITI training for human subjects' research.

24. References

1. Mahon NE, Yarcheski A, Yarcheski TJ. Differences in Social Support and Loneliness in Adolescents According to Developmental Stage and Gender. *Public Health Nurs*. 1994;11(5):361-368. doi:10.1111/j.1525-1446.1994.tb00199.x
2. Bruce LDH, Wu JS, Lustig SL, Russell DW, Nemecek DA. Loneliness in the United States: A 2018 National Panel Survey of Demographic, Structural, Cognitive, and Behavioral Characteristics. *Am J Heal Promot*. 2019;33(8):1123-1133. doi:10.1177/0890117119856551
3. Vanhalst J, Rassart J, Luyckx K, et al. Trajectories of Loneliness in Adolescents With Congenital Heart Disease: Associations With Depressive Symptoms and Perceived Health.

Published online 2013. doi:10.1016/j.jadohealth.2013.03.027

4. McGinty EE, Presskreischer R, Han H, Barry CL. Psychological Distress and Loneliness Reported by US Adults in 2018 and April 2020. *JAMA - J Am Med Assoc*. 2020;324(1):93-94. doi:10.1001/jama.2020.9740
5. Xia N, Li H. Loneliness, Social Isolation, and Cardiovascular Health. *Antioxidants Redox Signal*. 2018;28(9):837-851. doi:10.1089/ars.2017.7312
6. Caspi A, Harrington HL, Moffitt TE, Milne BJ, Poulton R. Socially isolated children 20 years later: Risk of cardiovascular disease. *Arch Pediatr Adolesc Med*. 2006;160(8):805-811. doi:10.1001/archpedi.160.8.805
7. Lacey RE, Kumari M, Bartley M. Social isolation in childhood and adult inflammation: Evidence from the National Child Development Study. *Psychoneuroendocrinology*. Published online 2014. doi:10.1016/j.psyneuen.2014.08.007
8. Heffner KL, Waring ME, Roberts MB, Eaton CB, Gramling R. Social isolation, C-reactive protein, and coronary heart disease mortality among community-dwelling adults. *Soc Sci Med*. 2011;72(9):1482-1488. doi:10.1016/j.socscimed.2011.03.016
9. Bu F, Zaninotto P, Fancourt D. Longitudinal associations between loneliness, social isolation and cardiovascular events. *Heart*. 2020;0:1-6. doi:10.1136/heartjnl-2020-316614
10. Valtorta NK, Kanaan M, Gilbody S, Ronzi S, Hanratty B. Loneliness and social isolation as risk factors for coronary heart disease and stroke. *Heart*. Published online 2016. doi:10.1136/heartjnl-2015-308790 T4 - Systematic review and meta-analysis of longitudinal observational studies Y3 - 22.04.2016 U6 - http://heart.bmj.com/content/early/2016/03/15/heartjnl-2015-308790.full.pdf#page=1&view=FitH M4 - Citavi
11. Yu B, Steptoe A, Chen L-J, Chen Y-H, Lin C-H, Ku P-W. Social Isolation, Loneliness, and All-Cause Mortality in Patients With Cardiovascular Disease. *Psychosom Med*. 2020;82(2):208-214. doi:10.1097/PSY.0000000000000777
12. Estévez-Báez M, Carricarte-Naranjo C, Jas-García JD, et al. Influence of Heart Rate, Age, and Gender on Heart Rate Variability in Adolescents and Young Adults. In: *Advances in Experimental Medicine and Biology*. Vol 1133. Springer New York LLC; 2019:19-33. doi:10.1007/5584_2018_292
13. Koenig J, Kemp AH, Beauchaine TP, Thayer JF, Kaess M. Depression and resting state heart rate variability in children and adolescents-A systematic review and meta-analysis. Published online 2016. doi:10.1016/j.cpr.2016.04.013
14. Paniccia M, Paniccia D, Thomas S, Taha T, Reed N. Clinical and non-clinical depression and anxiety in young people: A scoping review on heart rate variability. Published online 2017. doi:10.1016/j.autneu.2017.08.008
15. Shahrestani S, Stewart EM, Quintana DS, Hickie IB, Guastella AJ. Heart rate variability during adolescent and adult social interactions: A meta-analysis. *Biol Psychol*. 2015;105:43-50. doi:10.1016/j.biopsycho.2014.12.012
16. Horsten M, Ericson M, Perski A, Wamala SP, Schenck-Gustafsson K, Orth-Gomér K. Psychosocial factors and heart rate variability in healthy women. *Psychosom Med*. 1999;61(1):49-57. doi:10.1097/00006842-199901000-00009
17. Cruz FC, Duarte JO, Leão RM, Hummel LFV, Planeta CS, Crestani CC. Adolescent

vulnerability to cardiovascular consequences of chronic social stress: Immediate and long-term effects of social isolation during adolescence. *Dev Neurobiol*. 2016;76(1):34-46. doi:10.1002/dneu.22297

18. Hamilton JL, Alloy LB. Atypical reactivity of heart rate variability to stress and depression across development: Systematic review of the literature and directions for future research. *Clin Psychol Rev*. 2016;50:67-79. doi:10.1016/j.cpr.2016.09.003
19. Moraes ÍAP, Silva TD, Massetti T, et al. Fractal correlations and linear analyses of heart rate variability in healthy young people with different levels of physical activity. *Cardiol Young*. 2019;29(10):1236-1242. doi:10.1017/S1047951119001793
20. Scott BG, Alfano CA, Russell JD, Weems CF. Heart rate variability and anxious arousal: Unique relations with sleep-related problems in stress-exposed adolescents. *Dev Psychobiol*. 2019;61(8):1180-1190. doi:10.1002/dev.21883
21. Rodríguez-Colón SM, He F, Bixler EO, et al. Sleep variability and cardiac autonomic modulation in adolescents - Penn State Child Cohort (PSCC) study. *Sleep Med*. 2015;16(1):67-72. doi:10.1016/j.sleep.2014.10.007
22. Holt-Lunstad J, Smith TB. Loneliness and social isolation as risk factors for CVD: implications for evidence-based patient care and scientific inquiry. *Heart*. 2016;102(13):987-989. doi:10.1136/heartjnl-2015-309242
23. Heron KE, Everhart RS, McHale SM, Smyth JM. Using Mobile-Technology-Based Ecological Momentary Assessment (EMA) Methods with Youth: A Systematic Review and Recommendations. *J Pediatr Psychol*. Published online 2017. doi:10.1093/jpepsy/jsx078
24. Brannon EE, Cushing CC, Crick CJ, Mitchell TB. The promise of wearable sensors and ecological momentary assessment measures for dynamical systems modeling in adolescents: a feasibility and acceptability study. *Transl Behav Med*. 2016;6(4):558-565. doi:10.1007/s13142-016-0442-4
25. Ridgers ND, McNarry MA, Mackintosh KA. Feasibility and Effectiveness of Using Wearable Activity Trackers in Youth: A Systematic Review. *JMIR mHealth uHealth*. 2016;4(4):e129. doi:10.2196/mhealth.6540
26. Scott JJ, Rowlands A V, Cliff DP, Morgan PJ, Plotnikoff RC, Lubans DR. Comparability and feasibility of wrist-and hip-worn accelerometers in free-living adolescents. *J Sci Med Sport*. 2017;20:1101-1106. doi:10.1016/j.jsams.2017.04.017
27. Mahon NE, Yarcheski A. The dimensionality of the UCLA loneliness scale in early adolescents. *Res Nurs Health*. 1990;13(1):45-52. doi:10.1002/nur.4770130108
28. Mahon NE, Yarcheski TJ, Yarcheski A. *Validation of the Revised UCLA Loneliness Scale for Adolescents*. Vol 18.; 1995.
29. Lee R, Robbins SB. Measuring Belongingness: The Social Connectedness and the Social Assurance Scales Social and Emotional Learning View project KAD Parenting View project. *Artic J Couns Psychol*. Published online 1995. doi:10.1037/0022-0167.42.2.232
30. Primack BA, Shensa A, Sidani JE, et al. Social Media Use and Perceived Social Isolation Among Young Adults in the U.S. *Am J Prev Med*. Published online 2017. doi:10.1016/j.amepre.2017.01.010
31. Irish LA, Kline CE, Rothenberger SD, et al. A 24-hour approach to the study of health behaviors: Temporal relationships between waking health behaviors and sleep. *Ann Behav Med*. Published online 2014. doi:10.1007/s12160-013-9533-3

32. Almeida DM, McGonagle K, King H. Assessing daily stress processes in social surveys by combining stressor exposure and salivary cortisol. *Biodemography Soc Biol*. Published online 2009. doi:10.1080/19485560903382338
33. Kessler RC, Andrews G, Colpe LJ, et al. Short screening scales to monitor population prevalences and trends in non-specific psychological distress. *Psychol Med*. Published online 2002. doi:10.1017/S0033291702006074
34. Yung S, Chen Y, Zawadzki MJ. Loneliness and psychological distress in everyday life among Latinx college students. *J Am Coll Heal*. Published online 2021. doi: 10.1080/07448481.2021.1927051
35. Albers G, McNally RJ, Heeren A, de Wit S, Fried EI. Social media and depression symptoms: A network perspective. *J Exp Psychol Gen*. 2019;148(8):1454-62. doi: 10.1037/xge0000528.
36. Bennett BL, Whisenhunt BL, Hudson DL, et al. Examining the impact of social media on mood and body dissatisfaction using ecological momentary assessment. *J Am Coll Heal*. Published online 2020. doi:10.1080/07448481.2019.1583236
37. Primack BA, Karim SA, Shensa A, Bowman N, Knight J, Sidani JE. Positive and Negative Experiences on Social Media and Perceived Social Isolation. *Am J Heal Promot*. 2019;33(6):859-868. doi:10.1177/0890117118824196
38. Hamburger M, Basile KC, Vivolo A. *Measuring Bullying Victimization, Perpetration, and Bystander Experiences: A Compendium of Assessment Tools.*; 2011.
39. Clark R, Coleman AP, Novak JD. Brief report: Initial psychometric properties of the everyday discrimination scale in black adolescents. *J Adolesc*. Published online 2004. doi:10.1016/j.adolescence.2003.09.004
40. Burke Harris N, Renschler T. *Center for Youth Wellness Adverse Childhood Experiences*.; 2015.
41. Boterhoven de Haan KL, Hafekost J, Lawrence D, Sawyer MG, Zubrick SR. Reliability and Validity of a Short Version of the General Functioning Subscale of the McMaster Family Assessment Device. *Fam Process*. Published online 2015. doi:10.1111/famp.12113
42. Fisher P, Desai P, Klotz J, et al. COVID-19 Experiences Scale (COVEX). Disaster Lit®a database of the U.S. National Library of Medicine. Published 2020. https://dr2.nlm.nih.gov/search/?q=&data_type=10&source=2258
43. Control C for D. Youth Risk Behavior Scale Survey. https://www.cdc.gov/healthyyouth/data/yrbs/pdf/2021/2021-YRBS-Standard-HS-Questionnaire.pdf
